# Lysyl oxidase-like 1 gene polymorphisms in Japanese patients with primary open angle glaucoma and exfoliation syndrome

**Published:** 2008-07-14

**Authors:** Fumihiko Mabuchi, Yoichi Sakurada, Kenji Kashiwagi, Zentaro Yamagata, Hiroyuki Iijima, Shigeo Tsukahara

**Affiliations:** 1 Department of Ophthalmology, University of Yamanashi, Yamanashi, Japan; 2 Department of Health Sciences, University of Yamanashi, Yamanashi, Japan

## Abstract

**Purpose:**

To assess whether lysyl oxidase-like 1 (*LOXL1*) polymorphisms are associated with primary open-angle glaucoma (POAG) and exfoliation syndrome (XFS).

**Methods:**

Japanese patients with POAG (n=213) or XFS (n=89) and 191 control subjects were analyzed for *LOXL1* polymorphisms (rs1048661: 758G/T, Arg141Leu and rs3825942: 794G/A, Gly153Asp). Demographic and clinical features of POAG patients and control subjects were compared in terms of the TT/GG compound genotype of rs1048661 and rs3825942.

**Results:**

There was a significant difference in the genotype frequencies between XFS patients and control subjects (p<0.0001). Frequencies of the T allele of rs1048661 and the G allele of rs3825942 were significantly higher in XFS patients than in control subjects (rs1048661: 99.4% versus 55.0%; rs3825942: 99.4% versus 85.3%; p<0.0001). Except for one who had the TG/AG compound genotype, all XFS patients had the TT/GG compound genotype. An almost 250 fold increase in the risk of XFS (p<0.0001; odds ratio: 252.2; 95% confidence interval: 32.7 to more than 1000) was found in patients with the TT/GG compound genotype compared to those without the genotype. There were no significant differences in the genotype and allele frequencies between POAG patients and control subjects. Furthermore, no significant differences were noted in the demographic and clinical features of POAG patients as well as control subjects with and without the TT/GG high-risk compound genotype.

**Conclusions:**

*LOXL1* polymorphisms were associated with XFS. However, the frequencies of the polymorphisms differed between Japanese and Caucasian XFS patients. These polymorphisms had no influence on the phenotypic features of POAG patients.

## Introduction

Exfoliation syndrome (XFS) is characterized by the accumulation of extracellular fibrillar materials in various organs of the body [1,2]. In the eye, exfoliation materials develop mainly on the lens surface, corneal endothelium, iris, trabecular meshwork, zonula, and ciliary body [3]. Characteristic tissue alterations are predisposed to a broad spectrum of intraocular complications including insufficient mydriasis, blood-aqueous barrier dysfunction, phacodonesis, and angle closure glaucoma [3,4]. It is said that XFS is one cause of open-angle glaucoma. Jeng et al. [5] reported that the 15 year risk of XFS conversion into exfoliation glaucoma (XFG) is approximately 60%. As XFG is characterized by rapid progression, high resistance to medical therapy, and poor prognosis [6], it is important to identify the exact composition of XFS materials as well as the molecular mechanism responsible for its excessive production and progressive accumulation.

Thorleifsson et al. [7], in 2007, demonstrated that two single nucleotide polymorphisms (SNPs), rs1048661 (R141L) and rs3825942 (G153D), in exon 1 of the lysyl oxidase-like 1 (*LOXL1*) gene confer risk of XFG, possibly through XFS, in patients from Iceland and Sweden. They reported that approximately 25% of the general population was homozygous for the highest risk haplotype and that the risk of XFG for those 25% was more than 700 times that of individuals carrying only low risk haplotypes. They also reported that the two SNPs in *LOXL1* were not associated with primary open-angle glaucoma (POAG). However, the *LOXL1* high risk genotype for XFS may affect the phenotypic features such as maximum intraocular pressure (IOP) of patients with POAG. In this study, we investigated whether the two SNPs in *LOXL1* were associated with XFS and POAG in the Japanese population. Furthermore, we also investigated in detail the association between the two SNPs and the phenotypic features of patients with POAG.

## Methods

### Subjects

Japanese patients with POAG or XFS including XFG were recruited from ophthalmology practices in the University of Yamanashi Hospital, Enzan Municipal Hospital, Uenohara City Hospital, and Oizumi Clinic in Yamanashi or Nagano Prefecture, which are all located in the central portion of Japan (mostly the same population as that previously reported from Japan [8]). A diagnosis of POAG was made when open angles were detected on gonioscopic examination and typical glaucomatous cupping of the optic disc with compatible visual field defects was detected by automated static perimetry (Humphrey Visual Field Analyzer 30–2, Humphrey Instruments, San Leandro, CA). Additionally, patients were diagnosed with POAG when at least one previous measurement of IOP with a Goldmann applanation tonometer exceeded 21 mmHg. A diagnosis of XFS was made when accumulation of abnormal microfibrillar deposits on the papillary ruff with or without a grayish central disc on the anterior lens capsule was detected in at least one eye. Patients were excluded if they had a history of eye surgery including laser treatment such as laser trabeculoplasty before the diagnosis of POAG or XFS. Control subjects recruited from the participating institutions were Japanese who were over 40 years of age, had an IOP below 21 mmHg, exhibited no XFS and no glaucomatous cupping of the optic disc (no thinning of disc rim and cup-to-disc ratio less than 0.4), and had no family history of glaucoma. All subjects received comprehensive ophthalmologic examinations including both slit lamp biomicroscopy and fundoscopy, and peripheral blood was collected. The study protocol was approved by the ethics committee of the University of Yamanashi, and informed consent was obtained from all study participants. The study was conducted in accordance with the Declaration of Helsinki.

### Genomic DNA genotyping

Genomic DNA was purified with a Flexi Gene® DNA Kit (Qiagen, Valencia, CA), and two nonsynonymous SNPs, rs1048661 (758G/T, Arg141Leu) and rs3825942 (794G/A, Gly153Asp), both located in exon 1 of *LOXL1*, were genotyped by restriction fragment length polymorphism assay. The following primers were used for amplification: forward primer: 5′-GCT GAT CCA GTG GGA GAA CAA-3′ and reverse primer: 5′-GGG CTG GTA GGG GTA GAT GA-3′.

**Figure 1:**
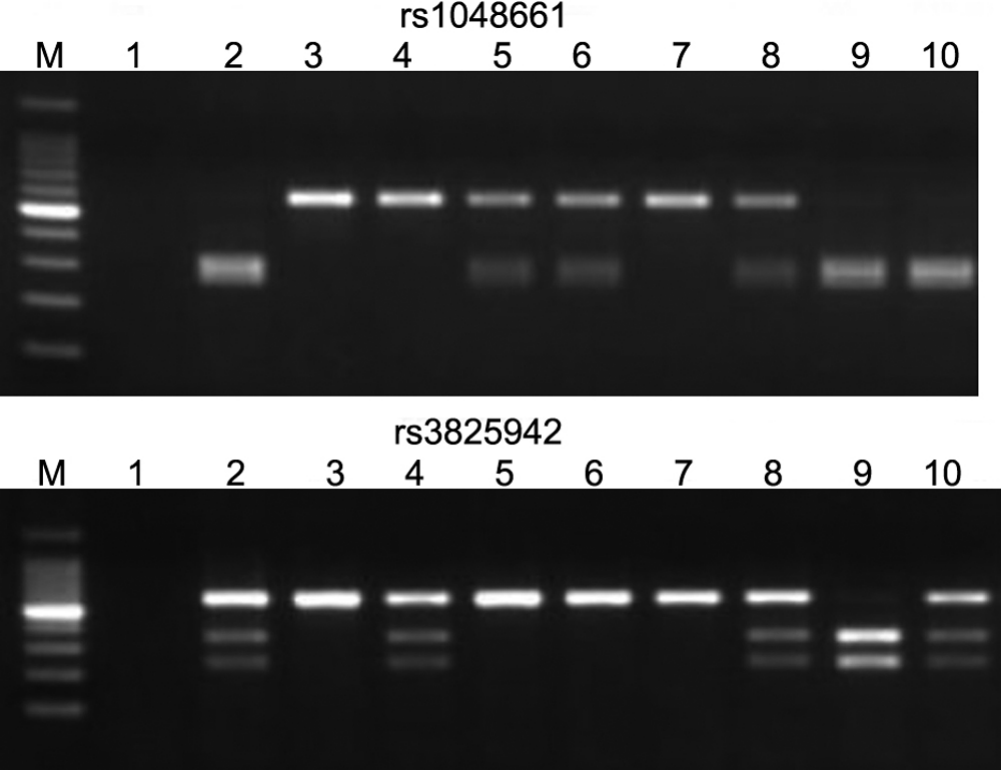
Electrophoresis of polymerase chain reaction-restriction fragment length polymorphism products for detection of nucleotide alterations of rs1048661 and rs3825942. DNA fragments with G at codon 141 (rs1048661) contained the Apa I restriction site, resulting in fragments of 304 bp+264 bp, while DNA fragments with T lacked the Apa I restriction site, resulting in a fragment of 568 bp. DNA fragments with A at codon 153 (rs3825942) contained the Hinf I restriction site, resulting in fragments of 335 bp+233 bp, while DNA fragments with G lacked the Hinf I restriction site, resulting in a fragment of 568 bp. Information for rs1048661: M; 100 bp marker, Lane 1; H_2_O (negative control), Lane 2; GG (positive control), Lane 3; TT (positive control), Lane 9 and 10; GG, Lane 5, 6, and 8; TG, Lane 4 and 7; TT. Information for rs3825942: M; 100 bp marker, Lane 1; H_2_O (negative control), Lane 2; AG (positive control) Lane 3; GG (positive control), Lane 5, 6, and 7; GG, Lane 4, 8, and 10; AG, Lane 9; AA.

The primers amplified the DNA region that spans both *LOXL1* polymorphic sites. Amplification product (5 μl) was digested with 4 units of Apa I (New England Biolabs, Beverly, MA) for 2 h at 25 °C to genotype rs1048661 or digested with 4 units of Hinf I (New England Biolabs, Beverly, MA) for 2 h at 37 °C to genotype rs3825942. DNA fragments with G at codon 141 (corresponding to an arginine residue) contained the Apa I restriction site, resulting in fragments of 304 bp+264 bp, while DNA fragments with T at codon 141 (corresponding to a leucine residue) lacked the Apa I restriction site, resulting in a fragment of 568 bp. DNA fragments with A at codon 153 (corresponding to an aspartic acid residue) contained the Hinf I restriction site, resulting in fragments of 335 bp+233 bp, while DNA fragments with G at codon 153 (corresponding to a glycine residue) lacked the Hinf I restriction site, resulting in a fragment of 568 bp. After the amplification products were loaded on 2% agarose gels and electrophoresed, the gels were stained with ethidium bromide and genotypes were obtained (Figure 1). To perform accurate genotyping, amplification products were accurately genotyped by direct sequencing and were used as positive controls for restriction fragment length polymorphism assay. Arg141Leu heterozygosity or Gly153Asp heterozygosity polymerase chain reaction products were inserted into a pGEM-T vector (Promega Corporation, San Luis Obispo, CA) by TA cloning. Plasmid DNA was amplified with the following primers: M13-forward primer: 5′-GTA AAA CGA CGG CCA G-3′ and M13-reverse primer: 5′-GGA AAC AGC TAT GAC CAT G-3′

After amplification, nucleotide sequences were determined with a Thermo Sequenase^TM^ Primer Cycle Sequencing Kit (GE Healthcare Bio-Sciences, Buckinghamshire, England) and 5′-Texas Red labeling M13-forward or M13-reverse primer. Sequence data were compared with the sequence registered in the NCBI GenBank (NM_005576), and 141Leu-153Gly homozygosity and 141Arg-153Asp homozygosity were identified and used as positive controls. Heterozygosity control was obtained by mixing these two homozygosity plasmids.

### Statistical analysis

Data were analyzed using SAS statistical software (version 9.1, SAS Institute Inc., Cary, NC). χ^2^ analysis of the Hardy–Weinberg equilibrium for *LOXL1* genotypes was performed for patients and control subjects. Genotype and allele frequency differences between POAG or XFS patients and control subjects were estimated by the χ^2^ test and Fisher’s exact test, respectively. Statistical analyses of the compound genotype of rs1048661 and rs3825942 were conducted using the χ^2^ test. We also used a logistic regression model to simultaneously study the effects of multiple variables when comparing XFS patients with control subjects. The predictor variables were age, gender, and TT/GG compound genotype of rs1048661 and rs3825942. The odds ratio of age is per year. We also compared demographic and clinical features including age at the time of blood sampling and diagnosis, gender, family history of glaucoma, refractive error, maximum IOP, and history of laser trabeculoplasty and glaucoma surgery of control subjects and POAG patients with and without the TT/GG compound genotype using the Student *t*-test for continuous variables and Fisher’s exact test for comparison of proportions. p<0.05 was considered to be statistically significant.

## Results

Two hundred and thirteen Japanese patients with POAG, 89 patients with XFS (67 of 89 patients were XFG), and 191 control subjects were enrolled in this study. The mean age at the time of blood sampling was 62.9±14.8 (standard deviation) years for POAG patients (range: 20–95 years), 76.5±6.6 years for XFS patients (range: 60–92 years), and 65.7±11.4 years for control subjects (range: 41–87 years). The mean age at the time of diagnosis was 54.4±15.3 years and 70.4±8.2 years for POAG and XFS patients, respectively. Genotype and allele frequencies of the two SNPs, rs1048661 and rs3825942, in POAG patients, XFS patients, and control subjects are shown in Table 1. These genotype and allele frequencies were in Hardy–Weinberg equilibrium in POAG and XFS patients and control subjects. Whereas no statistically significant differences in genotype and allele frequencies were found between POAG patients and control subjects, a significant difference in genotype frequencies of rs1048661 and rs3825942 was noted between XFS patients and control subjects (p<0.0001, χ^2^ test). Frequencies of the T allele of rs1048661 and the G allele of rs3825942 were significantly higher in XFS patients than in control subjects (rs1048661: 99.4% versus 55.0%, rs3825942: 99.4% versus 85.3%, p<0.0001, Fisher’s exact test). The compound genotype frequencies of these two SNPs in POAG patients, XFS patients, and control subjects are shown in Table 2. Except one who had the TG/AG compound genotype, all XFS patients had the TT/GG compound genotype of rs1048661 and rs3825942. A significant difference in compound genotype frequencies was noted between XFS patients and control subjects (p<0.0001, χ^2^ test). After adjusting for age and gender, an almost 250 fold increase in risk of XFS (p<0.0001, odds ratio: 252.2, 95% confidence interval: 32.7 to more than 1000) was found in patients with the TT/GG compound genotype compared to those with no such genotype. No significant differences were noted in the demographic and clinical features of POAG patients as well as control subjects with and without the TT/GG compound genotype (Table 3).

**Table 1:** Genotype and allele frequencies of rs1048661 and rs3825942 in patients with primary open angle glaucoma and exfoliation syndrome and control subjects.

**SNP**	**Genotype/ Allele**	**Control (n=191)**	**POAG (n=213)**	p value*	XFS** (n=89)	p value*
rs1048661 **(Arg141Leu)**	**Genotype**					
	TT (Leu/Leu)	59 (30.9)	59 (27.7)		88 (98.9)	
	TG (Leu/Arg)	92 (48.2)	107 (50.2)	0.78	1 (1.1)	<0.0001
	GG (Arg/Arg)	40 (20.9)	47 (22.1)		0 (0)	
	**Allele**					
	T	210 (55.0)	225 (52.8)	0.57	177 (99.4)	<0.0001
	G	172 (45.0)	201 (47.2)		1 (0.6)	
rs3825942 **(Gly1153Asp)**	**Genotype**					
	AA (Asp/Asp)	8 (4.2)	6 (2.8)		0 (0)	
	AG (Asp/Gly)	40 (20.9)	52 (24.4)	0.57	1 (1.1)	<0.0001
	GG (Gly/Gly)	143 (74.9)	155 (72.8)		88 (98.9)	
	**Allele**					
	A	56 (14.7)	64 (15.0)	0.92	1 (0.6)	<0.0001
	G	326 (85.3)	362 (85.0)		177 (99.4)	

A significant difference in genotype frequencies of rs1048661 and rs3825942 was noted between XFS patients and control subjects. Frequencies of the T allele of rs1048661 and the G allele of rs3825942 were significantly higher in XFS patients than in control subjects. Data are given as numbers (percentage). The asterisk indicates that the χ^2^ test or the Fisher's exact test was done. The double asterisk indicates that 67of 89 XFS patients had exfoliation glaucoma. POAG: primary open-angle glaucoma, XFS: exfoliation syndrome.

**Table 2:** rs1048661 and rs3825942 compound genotypes in patients with primary open angle glaucoma and exfoliation syndrome and control subjects.

**Genotype**rs1048661**T/G/**rs3825942**A/G**	**Control(n=191)**	**POAG (n=213)**	p value*	XFS** (n=89)	p value*
GG/AA	8 (4.2)	6 (2.8)		0 (0)	
GG/AG	14 (7.3)	15 (7.0)		0 (0)	
GG/GG	18 (9.4)	26 (12.2)	0.76	0 (0)	< 0.0001
TG/AG	26 (13.6)	37 (17.4)		1 (1.1)	
TG/GG	66 (34.6)	70 (32.9)		0 (0)	
TT/GG	59 (30.9)	59 (27.7)		88 (98.9)	

A significant difference in compound genotype frequencies was noted between XFS patients and control subjects. Data are given as numbers (percentage). The asterisk indicates that the χ^2^ test was done. The double asterisk indicates that 67 of 89 XFS patients had exfoliation glaucoma. POAG: primary open-angle glaucoma, XFS: exfoliation syndrome.

**Table 3:** Demographic and clinical features of control subjects and primary open angle glaucoma patients with or without TT/GG compound genotype of rs1048661 and rs3825942 in *LOXL1*.

	**Control**			**POAG**		
**Feature**	**TT/GG compound genotype**			**TT/GG compound genotype**		
	**+ (n=59)**	**- (n=132)**	p value*	**+ (n=59)**	**- (n=154)**	p value*
**Age at blood sampling (years)**	64.0 ± 12.2	66.5 ± 11.0	0.16	63.2±14.9	62.8±14.8	0.88
**Age at diagnosis (years)**	-	-	-	56.3±15.4	53.7±15.2	0.28
**Male gender, n (%)**	22 (37.3)	48 (36.4)	>0.99	40 (67.8)	89 (57.8)	0.21
**Familial history of glaucoma, n (%)**	0 (0)	0 (0)	1	15 (25.4)	47 (30.5)	0.5
**Refractive error (diopter)**	−0.45±2.2	−0.31±2.1	0.67	−2.2±3.2	−2.1±2.9	0.84
**Maximum IOP (mmHg)**	15.2 ± 2.6	14.9±2.8	0.55	27.4±6.0	28.9±9.9	0.27
**History of laser trabeculoplasty, n (%)**	-	-	-	10 (16.9)	26 (16.9)	>0.99
**History of glaucoma surgery, n (%)**	-	-	-	18 (30.5)	48 (31.2)	>0.99

No significant differences were noted in the demographic and clinical features of POAG patients as well as control subjects with and without the TT/GG compound genotype. Continuous variables are expressed as means±standard deviation. The asterisk indicates that the Student *t*-test or the Fisher's exact test was done. *LOXL1*: lysyl oxidase-like 1, POAG: primary open angle glaucoma, IOP: intraocular pressure.

## Discussion

The pathology of XFS is characterized by chronic accumulation of abnormal fibrillar material. The current pathogenetic concept describes XFS as an elastic microfibrillopathy (abnormal aggregation of elastin microfibrillar components) [6]. *LOXL1* is a member of the lysyl oxidase family of proteins that catalyzes oxidative deamination of lysine residues of tropoelastin, which leads to their spontaneous cross-linking with consequent formation of elastin polymer fibers [9,10]. Thorleifsson et al. [7] reported that allele G was the risk allele of rs1048661 and rs3825942 for XFS, the (G, G) haplotype had the highest risk, and the risk G allele of rs1048661 was associated with low expression of *LOXL1* mRNA, which suggests that low *LOXL1* expression predisposes to XFS. Similar frequencies of *LOXL1* SNP alleles, genotypes, and haplotypes were also reported in Caucasian Australian [11], American [12-16], and European [16] populations as well as the Indian [17] population. In this study, two SNPs, rs1048661 and rs3825942, were associated with XFS. However, the allele, genotype, and compound genotype frequencies of these two SNPs differed between our XFS patients and Caucasian XFS patients, as Hayashi et al. [8] reported. We found that, except one who had the TG/AG compound genotype, all XFS patients had the TT/GG compound genotype of rs1048661 and rs3825942 and that allele T and not G was the risk allele of rs1048661. It is difficult to provide an explanation why allele T of rs1048661 is associated with XFS in the Japanese population alone. However, these data suggest that rs1048661 (R141L) may be little related to the occurrence of XFS, and the reduced *LOXL1* expression by allele G of rs1048661 may be a small change and not necessarily biologically meaningful as Thorleifsson et al. [7] described. In the Japanese population, it is considered that two copies of haplotype (T, G) are in principle necessary for the occurrence of XFS, and the T allele of rs1048661 plays an important role as a risk allele. In our control subjects, the frequency of T allele of rs1048661 was higher than that of G allele, contrary to the reported allele frequencies in Caucasian populations [7,11-16]. The prevalence of XFS in the Japanese population over 60 years of age is 2.1% [18], which is not high compared to other ethnic populations such as the Nordic population [19]. The characteristic allele frequencies of *LOXL1* may affect the low prevalence of XFS in the Japanese population. It remains to be determined whether there are subtypes of XFS with different patterns of manifestation. XFS patients in this study presented with either unilateral or bilateral involvement with or without elevated IOP. It seems difficult to explain these phenotype differences on the basis of the genotypes of *LOXL1* because most XFS patients had the same compound genotype. Several susceptibility loci [20-22] and other susceptibility genes [23,24] for XFS have been reported. Susceptibility genes without *LOXL1* may be associated with the phenotype differences in patients with XFS.

Signs of XFS often appear later in eyes initially diagnosed as having POAG. It is not clear whether this reflects an inaccurate clinical diagnosis or a coincidence of two different conditions. By thorough biomicroscopic examination, a diffuse-matte homogeneous film that consists of a layer of microfibrils can be observed on the entire surface of the anterior lens capsule before the formation of typical XFS deposits [3]. A prospective 10 year follow up study involving patients with clinically unilateral XFS showed that glaucoma may develop in the contralateral eye before the emergence of any signs of clinical XFS [25], indicating that exfoliation materials that form before clinically typical XFS deposits may cause open-angle glaucoma. In this study, although no statistically significant differences in genotype and allele frequencies were found between POAG patients and control subjects as previous studies have reported [7,15,26], approximately 30% of POAG patients had the TT/GG high-risk compound genotype for XFS in *LOXL1*. It was also found that exfoliation materials that form before any clinical signs of XFS may affect phenotypic features including maximum IOP in patients with POAG. However, as shown in Table 3, there were no significant differences in demographic and clinical features of POAG patients as well as control subjects with and without the TT/GG high-risk compound genotype, and this high-risk compound genotype was not associated with phenotypic features in patients with POAG. These results do not support the idea that exfoliation materials formed before any clinical signs of XFS are a cause of open-angle glaucoma and may indicate that exfoliation materials have little or no influence on the occurrence of POAG. In patients with the TT/GG high-risk compound genotype, mean ages at the time of blood sampling in control subjects and POAG patients were significantly younger than that in XFS patients. Some of these control subjects and POAG patients with the TT/GG compound genotype may present with clinically apparent XFS in the future because XFS is an age-related disorder.

In conclusion, *LOXL1* polymorphisms were associated with XFS. However, the allele, genotype, and compound genotype frequencies of these two SNPs differed between Japanese XFS patients and Caucasian XFS patients. These polymorphisms had no influence on the phenotypic feature of POAG patients. Further studies in the other ethnic populations should be performed to clarify how changes of *LOXL1* lead to XFS.

## References

[1] Streeten BW, Li ZY, Wallace RN, Eagle RC, Keshgegian AA (1992). Pseudoexfoliative fibrillopathy in visceral organs of a patient with pseudoexfoliation syndrome.. Arch Ophthalmol.

[2] Schlotzer-Schrehardt UM, Koca MR, Naumann GO, Volkholz H (1992). Pseudoexfoliation syndrome. Ocular manifestation of a systemic disorder?. Arch Ophthalmol.

[3] Naumann GO, Schlotzer-Schrehardt U, Kuchle M (1998). Pseudoexfoliation syndrome for the comprehensive ophthalmologist. Intraocular and systemic manifestations.. Ophthalmology.

[4] Ritch R, Schlotzer-Schrehardt U (2001). Exfoliation syndrome.. Surv Ophthalmol.

[5] Jeng SM, Karger RA, Hodge DO, Burke JP, Johnson DH, Good MS (2007). The risk of glaucoma in pseudoexfoliation syndrome.. J Glaucoma.

[6] Schlotzer-Schrehardt U, Naumann GO (2006). Ocular and systemic pseudoexfoliation syndrome.. Am J Ophthalmol.

[7] Thorleifsson G, Magnusson KP, Sulem P, Walters GB, Gudbjartsson DF, Stefansson H, Jonsson T, Jonasdottir A, Jonasdottir A, Stefansdottir G, Masson G, Hardarson GA, Petursson H, Arnarsson A, Motallebipour M, Wallerman O, Wadelius C, Gulcher JR, Thorsteinsdottir U, Kong A, Jonasson F, Stefansson K (2007). Common sequence variants in the LOXL1 gene confer susceptibility to exfoliation glaucoma.. Science.

[8] Hayashi H, Gotoh N, Ueda Y, Nakanishi H, Yoshimura N (2008). Lysyl oxidase-like 1 polymorphisms and exfoliation syndrome in the Japanese population.. Am J Ophthalmol.

[9] Liu X, Zhao Y, Gao J, Pawlyk B, Starcher B, Spencer JA, Yanagisawa H, Zuo J, Li T (2004). Elastic fiber homeostasis requires lysyl oxidase-like 1 protein.. Nat Genet.

[10] Lucero HA, Kagan HM (2006). Lysyl oxidase: an oxidative enzyme and effector of cell function.. Cell Mol Life Sci.

[11] Hewitt AW, Sharma S, Burdon KP, Wang JJ, Baird PN, Dimasi DP, Mackey DA, Mitchell P, Craig JE (2008). Ancestral LOXL1 variants are associated with pseudoexfoliation in Caucasian Australians but with markedly lower penetrance than in Nordic people.. Hum Mol Genet.

[12] Fingert JH, Alward WL, Kwon YH, Wang K, Streb LM, Sheffield VC, Stone EM (2007). LOXL1 mutations are associated with exfoliation syndrome in patients from the Midwestern United States.. Am J Ophthalmol.

[13] Yang X, Zabriskie NA, Hau VS, Chen H, Tong Z, Gibbs D, Farhi P, Katz BJ, Luo L, Pearson E, Goldsmith J, Ma X, Kaminoh Y, Chen Y, Yu B, Zeng J, Zhang K, Yang Z (2008). Genetic association of LOXL1 gene variants and exfoliation glaucoma in a Utah cohort.. Cell Cycle.

[14] Challa P, Schmidt S, Liu Y, Qin X, Vann RR, Gonzalez P, Allingham RR, Hauser MA (2008). Analysis of LOXL1 polymorphisms in a United States population with pseudoexfoliation glaucoma.. Mol Vis.

[15] Fan BJ, Pasquale L, Grosskreutz CL, Rhee D, Chen T, DeAngelis MM, Kim I, del Bono E, Miller JW, Li T, Haines JL, Wiggs JL (2008). DNA sequence variants in the LOXL1 gene are associated with pseudoexfoliation glaucoma in a U.S. clinic-based population with broad ethnic diversity.. BMC Med Genet.

[16] Aragon-Martin JA, Ritch R, Liebmann J, O'Brien C, Blaaow K, Mercieca F, Spiteri A, Cobb CJ, Damji KF, Tarkkanen A, Rezaie T, Child AH, Sarfarazi M (2008). Evaluation of LOXL1 gene polymorphisms in exfoliation syndrome and exfoliation glaucoma.. Mol Vis.

[17] Ramprasad VL, George R, Soumittra N, Sharmila F, Vijaya L, Kumaramanickavel G (2008). Association of non-synonymous single nucleotide polymorphisms in the LOXL1 gene with pseudoexfoliation syndrome in India.. Mol Vis.

[18] Yamamoto T, Iwase A, Araie M, Suzuki Y, Abe H, Shirato S, Kuwayama Y, Mishima HK, Shimizu H, Tomita G, Inoue Y, Kitazawa Y, Tajimi Study Group, Japan Glaucoma Society. (2005). The Tajimi Study report 2: prevalence of primary angle closure and secondary glaucoma in a Japanese population.. Ophthalmology.

[19] Ringvold A (1999). Epidemiology of the pseudo-exfoliation syndrome.. Acta Ophthalmol Scand.

[20] Kozobolis VP, Detorakis ET, Sourvinos G, Pallikaris IG, Spandidos DA (1999). Loss of heterozygosity in pseudoexfoliation syndrome.. Invest Ophthalmol Vis Sci.

[21] Zalewska R, Pepinski W, Smolenska-Janica D, Mariak Z, Proniewska-Skretek E, Skawronska M, Janica J (2003). Loss of heterozygosity in patients with pseudoexfoliation syndrome.. Mol Vis.

[22] Lemmela S, Forsman E, Sistonen P, Eriksson A, Forsius H, Jarvela I (2007). Genome-wide scan of exfoliation syndrome.. Invest Ophthalmol Vis Sci.

[23] Yilmaz A, Tamer L, Ates NA, Camdeviren H, Degirmenci U (2005). Effects of apolipoprotein E genotypes on the development of exfoliation syndrome.. Exp Eye Res.

[24] Ritland JS, Utheim TP, Utheim OA, Espeseth T, Lydersen S, Semb SO, Rootwelt H, Elsas T (2007). Effects of APOE and CHRNA4 genotypes on retinal nerve fibre layer thickness at the optic disc and on risk for developing exfoliation syndrome.. Acta Ophthalmol Scand.

[25] Puska PM (2002). Unilateral exfoliation syndrome: conversion to bilateral exfoliation and to glaucoma: a prospective 10-year follow-up study.. J Glaucoma.

[26] Chakrabarti S, Rao KN, Kaur I, Parikh RS, Mandal AK, Chandrasekhar G, Thomas R (2008). The LOXL1 gene variations are not associated with primary open-angle and primary angle-closure glaucomas.. Invest Ophthalmol Vis Sci.

